# A novel chalcone derivative which acts as a microtubule depolymerising agent and an inhibitor of P-gp and BCRP in *in-vitro *and *in-vivo *glioblastoma models

**DOI:** 10.1186/1471-2407-9-242

**Published:** 2009-07-20

**Authors:** Ahcene Boumendjel, Anne McLeer-Florin, Pierre Champelovier, Diane Allegro, Dima Muhammad, Florence Souard, Madiha Derouazi, Vincent Peyrot, Bertrand Toussaint, Jean Boutonnat

**Affiliations:** 1 CNRS, UMR 5063, Bâtiment E, Pôle Chimie, Grenoble, F-38700 France; 2 RFMQ, TIMC, IMAG, CNRS, UMR 5525, Bâtiment Jean Roget, Faculté de Médecine, Grenoble, F-38700 France; 3 GREPI-THEREX, TIMC, IMAG, CNRS, UMR 5525, Bâtiment Jean Roget, Faculté de Médecine, Grenoble, F-38700 France; 4 CRO2, Inserm U911, Aix-Marseille Université, Faculté de Pharmacie de Marseille, 27 bd Jean Moulin, Marseille, F-13005 France; 5 CHRU Grenoble, Hôpital Michallon, Département d'Anatomie et Cytologie Pathologiques (DACP), Grenoble, F-38000 France; 6 CHRU Grenoble, Hôpital Michallon, Département de Biologie et Pathologie de la Cellule (DBPC), Grenoble, F-38000 France

## Abstract

**Background:**

Over the past decades, in spite of intensive search, no significant increase in the survival of patients with glioblastoma has been obtained. The role of the blood-brain barrier (BBB) and especially the activity of efflux pumps belonging to the ATP Binding Cassette (ABC) family may, in part, explain this defect.

**Methods:**

The *in-vitro *activities of JAI-51 on cell proliferation were assessed by various experimental approaches in four human and a murine glioblastoma cell lines. Using drug exclusion assays and flow-cytometry, potential inhibitory effects of JAI-51 on P-gp and BCRP were evaluated in sensitive or resistant cell lines. JAI-51 activity on *in-vitro *microtubule polymerization was assessed by tubulin polymerization assay and direct binding measurements by analytical ultracentrifugation. Finally, a model of C57BL/6 mice bearing subcutaneous GL26 glioblastoma xenografts was used to assess the activity of the title compound *in vivo*. An HPLC method was designed to detect JAI-51 in the brain and other target organs of the treated animals, as well as in the tumours.

**Results:**

In the four human and the murine glioblastoma cell lines tested, 10 μM JAI-51 inhibited proliferation and blocked cells in the M phase of the cell cycle, via its activity as a microtubule depolymerising agent. This ligand binds to tubulin with an association constant of 2 × 10^5 ^M^-1^, overlapping the colchicine binding site. JAI-51 also inhibited the activity of P-gp and BCRP, without being a substrate of these efflux pumps. These *in vitro *studies were reinforced by our *in vivo *investigations of C57BL/6 mice bearing GL26 glioblastoma xenografts, in which JAI-51 induced a delay in tumour onset and a tumour growth inhibition, following intraperitoneal administration of 96 mg/kg once a week. In accordance with these results, JAI-51 was detected by HPLC in the tumours of the treated animals. Moreover, JAI-51 was detected in the brain, showing that the molecule is also able to cross the BBB.

**Conclusion:**

These *in vitro *and *in vivo *data suggest that JAI-51 could be a good candidate for a new treatment of tumours of the CNS. Further investigations are in progress to associate the title compound chemotherapy to radiotherapy in a rat model.

## Background

Glioblastoma represents the most common type of primary tumours of the central nervous system (CNS) [1], and has a poor prognosis (less than 12 months), requiring a multidisciplinary approach, including surgery, radiotherapy and chemotherapy [2]. The use of common anticancer drugs is hampered by the presence of the blood-brain barrier (BBB), causing poor distribution of these agents. This restrictive action of the BBB has been linked to the presence on the brain endothelial cells of drug efflux transport systems, especially transporters belonging to the ATP Binding Cassette (ABC) family [3,4], out of which two have been shown to have a functional importance *in vivo *on the BBB: the P-glycoprotein (P-gp/ABCB1) and the Breast Cancer Resistance Protein (BCRP/ABCG2) [5-8].

One way of efficiently treating glioblastoma is to identify bifunctional molecules able not only to block glioblastoma cells proliferation, but also to cross the BBB without being effluxed by ABC proteins. In searching for such molecules, we targeted the naturally occurring flavonoids and their derivatives [9]. We have already described the potential of flavonoid derivatives as antimitotics and as multidrug reversers [10-12]. In the present report, we describe the biological activities of a new derivative named JAI-51. This compound was identified by screening of a large series of chalcones and was optimized to obtain the best biological activity.

## Methods

### Chemical synthesis of JAI-51

**Figure 1:**
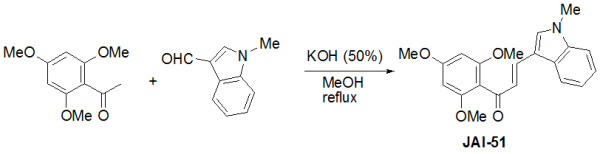
**Chemical synthesis of JAI-51**. See methods section for details.

The pharmacophore of the title compound was identified through a screening process. The optimization phase, which deals with identification of the optimal substituteds, led to the identification of JAI-51. The access to the target compound was achieved in one step starting from 2',4',6'-trimethoxyacetophenone (Figure 1). The latter was condensed with 1-methylindolyl-3-carboxaldehyde in the presence of KOH in a mixture of H_2_O:MeOH [12].

### In vitro studies

#### Culture of glioblastoma cell lines

Human glioblastoma derived cell lines U118, U138, U373 and LN229 were generously provided by Pr F. Berger (INSERM U318). These four cell lines as well as HCT116S and R, K562S and R, and the mouse glioma cell line GL26, were maintained in RPMI 1640 (U118, U138, U373, LN229) or DMEM (GL26) medium with 10% (v/v) inactivated fetal calf serum (FCS) (Gibco BRL, Eragny, France), antibiotics (penicillin 100 IU.ml^-1 ^and streptomycin 100 μg.ml^-1^), and L glutamine (2 mM) (Roche, Meylan, France) at 37°C in a humidified atmosphere with 5% CO2. In induced cultures, cells were seeded at 0.3 × 10^6 ^cells ml^-1 ^for 24 h before the addition of various doses of JAI-51 (1 μM to 10 μM) or DMSO (vehicle/0.1%) for various times (24 to 48 hours). At the indicated time, cells were trypsinized then non-adherent and adherent trypsinized cells were used for the determination of cell proliferation, cell cycle and apoptosis.

#### Proliferation, apoptosis and cell cycle analyses

The number of total and viable cells was determined using Trypan blue (0.4%) exclusion in triplicate and the mean value determined was then confirmed by MTT (3-(4,5-Dimethylthiazol-2-yl)-2,5-Diphenyltetrazolium Bromide) assay, as described by the manufacturer (Sigma Aldrich, l'Isle d'Abeau, France) by using 1 to 10 μM JAI-51 for 24 and 48 hours. Cell DNA content was analyzed using the CycleTestTM PLUS/DNA reagent kit (BD Sciences, San Jose, CA). Data were collected and analyzed on a FACSCalibur flow-cytometer (Becton Dickinson, La Jolla, CA) using CELLQUEST PROTM software (BD Sciences, Le Pont de Claix France). Apoptosis was confirmed using the Annexin V-FITC/PI method (Vybrant™ Apoptosis Assay kit, Molecular Probes, Eugene, OR) by flow-cytometry. The role of Caspase-3 in the apoptotic process was determined by the Caspase-3 Fluorimetric Assay (R&D Systems, Mineapolis, MN) as described by the manufacturer.

#### Determination of the mitotic index

At 24 hours induction time, tumour cells were trypsinized, cytocentrifuged (Cytospin, Shandon, Pittsburgh, PA) and stained using the Papanicolaou procedure. The mitotic index and the number of polynucleated cells were then determined on 300 cells with conventional morphology analysis using a Zeiss microscope (Oberkochen, Germany).

#### In-vitro evaluation of JAI-51 on BCRP and P-gp activities

The accumulation studies of mitoxantrone (BCRP substrate) and daunorubicin (P-gp substrate) were performed using wild type (sensitive) HCT116 cell line (HCT116S) which does not express BCRP, and the BCRP-transfected resistant form HCT116R, and on wild-type (sensitive) K562S or P-gp expressing (resistant) K562R cell lines [10,11]. Cells grown in RPMI 1640 medium with 10% FCS were trypsinized (HCT116), washed twice then re-suspended in RPMI 1640 medium with 10% FCS to obtain a density of 10^6 ^cells/ml. JAI-51 at various concentrations (10^-7 ^to 10^-5 ^M) or the BCRP and P-gp inhibitor cyclosporine A (1 μM final concentration) was added to 1 ml of cells and incubated for 15 min at 37°C, followed by addition of mitoxantrone (final concentration 3 μM) or daunorubicin (final concentration 1 μM). After 30 min of incubation at 37°C, 4 ml of ice-cold PBS were added to stop drug accumulation and fluorescence was quantified by flow-cytometry. Mitoxantrone and daunorubicin accumulation studies were also performed on the glioblastoma cell lines used in our study, in order to test for the presence of functional P-gp and/or BCRP efflux pumps on these cell lines.

#### Evaluation of JAI-51 as a potential substrate of P-gp and/or BCRP

HCT116/S and R, K562/S and R exponentially growing cells were incubated for 24 hours in the presence of DMSO (vehicle/0.1%) or JAI-51 (10 μM). Cell cycle analyses were done as described above.

#### In vitro tubulin polymerization assay and direct binding measurement

Tubulin was purified from soluble lamb brain homogenate by ammonium sulfate fractionation and ion exchange chromatography according to the published method [13-17].

Microtubule assembly was assayed in 20 mM sodium phosphate (NaPi), 10 mM MgCl_2_, 1 mM EGTA, 3.4 M glycerol, and 0.1 mM GTP, pH = 6.5, in the presence of various concentrations of JAI-51 (0–15 μM). The polymerization reaction (15 μM tubulin) was started by increasing the temperature to 37°C and the mass of polymer formed was monitored in thermostated cuvettes by measuring turbidity at 350 nm in a Beckman DU 7400 spectrophotometer [13]. The concentration of JAI-51 (stock solution in DMSO) was measured spectrophotometrically with an extinction coefficient ε_392 nm _= 27,000 M^-1^.cm^-1^.

The sedimentation of tubulin and tubulin-colchicine was analyzed, without and with various concentrations of JAI-51 (2.5–36 μM) in PG buffer (NaPi 20 mM, GTP 10^-4 ^M, pH = 7.0). The experiments were carried out at 40,000 rpm and 20°C in a Beckman Optima XL-A analytical ultracentrifuge equipped with absorbance optics, using an eight holes An50Ti rotor and 1.2 cm Epon double-sector centrepieces. Data were acquired in continuous mode at 392 nm, the maximum wavelength of JAI-51 (ε_392 nm _= 18,100 M^-1 ^cm^-1^, solution in aqueous neutral buffer). Apparent sedimentation coefficients were determined by the sedimentation coefficient distribution C(S) generated by SEDFIT program [18]. These analyses allowed us to quantify the bound ligand concentration by measuring the area under the tubulin sedimenting peak (5.4S) and the free concentration by measurement of the baseline (which represents the ligand that does not sediment, *i.e*, the free ligand). For various concentrations of JAI-51, bound ligand divided by total tubulin (B) was plotted versus free ligand (L) concentrations and the apparent association constant, K and the stoichiometry (n) were determined using the following equation:

### In vivo studies

#### Animals

C57BL/6 mice 5 to 6 weeks old with a weight of 20 ± 2 g were obtained from Elevage Janvier (Le Genest St Isle, France) and kept under pathogen-free conditions in the animal facility of the University Joseph Fourier. All animal experiments were approved by the Animal Experiment Committee of the University and were performed in accordance with institutional and national guidelines.

#### Treatment of subcutaneously implanted GL26 tumors

GL26 cells (1 × 10^5 ^cells in 100 μl PBS) were implanted subcutaneously in the left flank of each mouse. JAI-51 treatment was delivered intraperitonealy (*ip*) in 100 μl PBS. One group received only the vehicle (control), one group was treated three times a week with 32 mg/kg JAI-51 (schedule N°1) and one group was treated once a week with 96 mg/kg JAI-51 (schedule N°2). The first injection was done 48 h after the tumour challenge. The mice were inspected each day to detect the presence of a palpable tumour. The tumour size was then assessed by caliper measurement 2, 4, 7 and 11 days after the apparition of the tumour and the mice were sacrificed when the tumour size reached 10 millimetres. The day of GL26 cells implantation was designated as day 0.

#### Detection of JAI-51 in mice after intraperitoneal administration

A sensitive and reliable high performance liquid chromatography method (HPLC) with UV detection, using a solid-phase extraction (SPE) was established for detection of JAI-51 in brain, liver, kidney and tumours of mice. Saline perfusion (wash-out) was used to reduce intravascular contamination before collecting organs that were immediately frozen at -80°C. After thawing, organs were grinded, diluted with water, applied to a solid-phase extraction C_18 _cartridge and extracted by acetonitrile. The samples were analyzed by HPLC using UV detection at 386 nm. The limit of detection (LOD with *S/N *= 3) for JAI-51 was 3.85 × 10^-7 ^M and the linearity of the calibration curves was prepared by fitting absorbance *vs*. amount of JAI-51 standards between 3.85 × 10^-7 ^M and 20 × 10^-7 ^M (measured 3 times). As a control, a known concentration of JAI-51 was analyzed in the same experimental conditions as the samples to be sure that the peak detected corresponded to JAI-51. The detection of the unmetabolized compound was performed on brain, liver and kidney of three untreated mice and on three mice treated with JAI-51 (schedules 1 and 2) two days after the injection. On tumours, the detection was done an hour and a half after the injection, on three untreated mice and on three mice treated with schedule 2, as this schedule was the one showing a significant decrease in the mean tumour volume compared to the control mice.

#### Data analysis

Data are expressed as the mean ± SD of 2 (*in vivo *studies) or at least 3 (*in vitro *studies) experiments. For statistical analysis of *in vitro *data, one-way ANOVA was performed to determine differences among all groups (*P *< 0.05), and the Bonferroni/Dunn posttest was performed to determine the significance of the differences between pairs of groups. *P *< 0.05 was considered significant. The statistical tests were performed on StatView software (version 5.0, SAS Institute, Inc., Cary, NC). The *in vivo *data were analyzed with GraphPad Prism (version 5, La Jolla, CA) and following one-way ANOVA analysis, Dunn's Multiple Comparison Test was used to determine the significance of the differences between each of the two treatment schedules versus the control group.

## Results

### Effects of JAI-51 on glioblastoma tumor cell lines

Using four human glioblastoma cell lines (U118, U138, U373 and LN229) and one mouse glioblastoma cell line (GL26), JAI-51 was tested at concentrations varying from 1 to 10 μM and for various time points. Four parameters were subsequently analyzed: cell proliferation, cell cycle, apoptosis and mitotic index.

**Figure 2:**
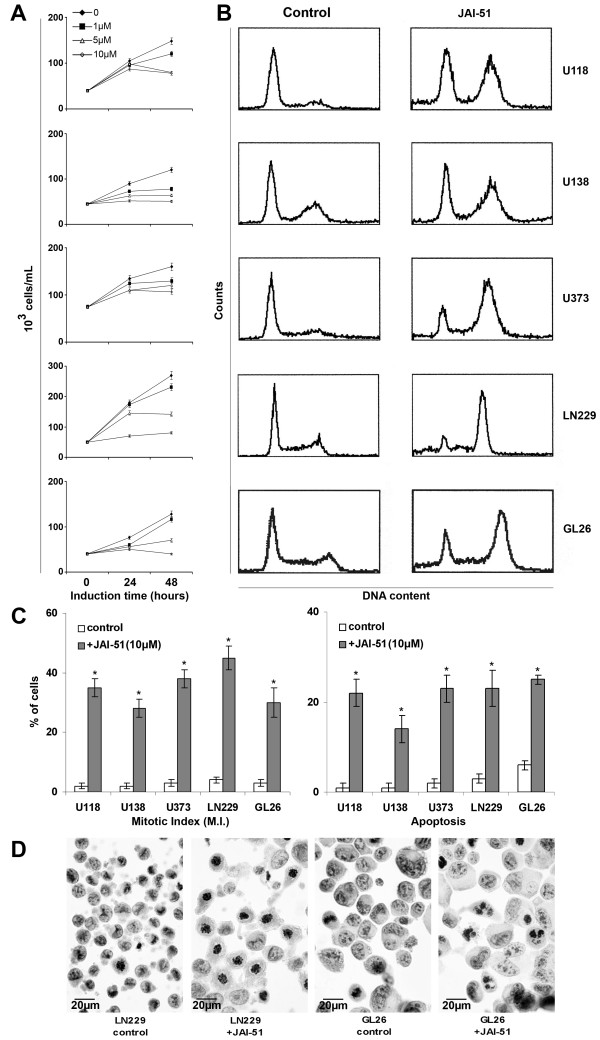
**Effects of JAI-51 on a panel of glioblastoma cell lines**. U118, U138, U373, LN229 cells were cultured for 24 h before the addition of JAI-51 (1 to 10 μM). Cells were used for either cell proliferation tests using MTT at the indicated times **(A) **or for cell cycle analysis at 24 h **(B)**. **(A) **After 24 h induction time with 10 μM JAI-51, the decrease in proliferation was statistically significant for all the cell lines tested compared to the untreated cells (*P *< 0.05 treated versus control), and after 48 h induction time, this decrease in proliferation was also significant with 5 μM JAI-51 for all the cell lines tested (*P *< 0.05 treated versus control). The results are expressed as the mean ± SD from 3 different experiments. Mitotic index (M.I.) **(C, left panel) **was determined at 24 h on slide after Papanicolaou staining, and apoptosis **(C, right panel) **was determined at 48 h using the Annexin V-FITC/PI method (* *P *< 0.05 treated versus control). **(D) **Photomicrographs of control LN229 and GL26 cells and cells treated with 10 μM JAI-51 for 24 h (LN229+JAI-51, GL26+JAI-51). Treated LN229 and GL26 cells displayed polylobated nuclei and most of the mitotic figures showed numerous abnormalities (complex spindle with several poles). (Magnification × 600).

The inhibition of cell proliferation as determined by the MTT test was time- and dose-dependent, with the concentrations of 5 μM and 10 μM of JAI-51 modulating the most the proliferation of the five cell lines (Figure 2/A). Indeed, after 24 h induction time with 10 μM JAI-51, the decrease in proliferation was statistically significant for all the cell lines tested compared to the untreated cells (*P *< 0.05 treated versus control), and after 48 h induction time, this decrease in proliferation was also significant with 5 μM JAI-51 for all the cell lines tested (*P *< 0.05 treated versus control).

At 24 hours induction time with 10 μM JAI-51, cell cycle distribution analysis showed a dramatic increase in the percentage of cells in the G2/M phase together with a decrease in G0/G1 and S populations (Figure 2/B and Table 1). Papanicolaou staining showed that the mitotic index (MI) increased in all the cell lines tested, suggesting an arrest in the M phase of the cell cycle (Figure 2/C**left panel **and **D**). Moreover, at 48 hours, the percentage of cells in the sub-G0/G1 phase (hypodiploid cells) dramatically increased, suggesting an apoptotic process (data not shown). To confirm this hypothesis, cells were treated with JAI-51 (10 μM for 48 hours) and the cell death process was investigated using the Annexin V-FITC/PI method and the Caspase-3 Fluorimetric Assay. The percentage of apoptotic cells and of hypodiploid cells increased after JAI-51 treatment in the five glioblastoma cell lines (Figure 2/C**right panel**). Apart from U138 cell line, the apoptotic process induced by 10 μM JAI-51 was Caspase-3 dependent (Table 2).

**Table 1:** Percentage of cells in the cell cycle phases after 24 hours incubation with 10 μM JAI-51

Cell cycle phase	U118C	U118T	U138C	U138T	U373C	U373T	LN229C	LN229T	GL26C	GL26T
G0/G1	60 ± 9	30 ± 5 *	54 ± 6	32 ± 6 *	54 ± 4	21 ± 3 *	53 ± 5	12 ± 4 *	55 ± 8	30 ± 5 *

S	18 ± 2	15 ± 3	10 ± 2	9 ± 2	20 ± 2	15 ± 2	18 ± 3	19 ± 4	22 ± 3	10 ± 3

G2/M	22 ± 3	55 ± 7 *	34 ± 7	58 ± 6 *	26 ± 3	64 ± 6 *	28 ± 3	68 ± 6 *	23 ± 4	60 ± 8 *

Glioblastoma cells were cultured for 24 hours without (C) or with 10 μM JAI-51 (T) before being stained with propidium iodide. DNA content was analyzed by flow-cytometry and the percentage of cells in each phase of the cell cycle was obtained by using Modfit program. Numbers ± SD indicate the percentage of cells and are the mean of 3 to 5 experiments. * *P *< 0.05 (treated versus control).

**Table 2:** Effect of JAI-51 on the apoptotic process in the glioblastoma cell lines.

Cell line	U118	U138	U373	LN229	GL26
Caspase-3 activity (A.U.)	13 ± 3*	6 ± 2	39 ± 3*	120 ± 25*	ND

Hypodiploid cells (%)	26 ± 10*	17 ± 1*	20 ± 2*	30 ± 5*	25 ± 5*

Apoptotic+necrotic cells (%)	21 ± 5*	20 ± 3*	34 ± 2*	31 ± 2*	25 ± 8*

Glioblastoma cells were cultured for 24 h before the addition of JAI-51 (10 μM) for 48 h. Caspase-3 activity, the percentage of cells with a DNA content <2 N (hypodiploid cells) and the percentage of apoptotic and necrotic cells were assessed. Results are the mean ± SD of 3 experiments. In the control cultures (not treated with JAI-51), Caspase activity was 4 ± 1 A.U. and the percentage of apoptotic cells was < 3% in all the cell lines. A.U.: arbitrary units; %: percentage of cells. * *P *< 0.05 (treated versus control).

### Effect of JAI-51 on MDR cancer cell lines

**Figure 3:**
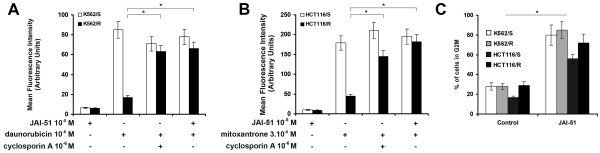
**Inhibition of P-gp and BCRP**. **(A) **Effects of JAI-51 on wild-type (S, sensitive) and on P-gp overexpressing (R, resistant) K562 cell lines, with or without daunorubicin (P-gP substrate) and/or cyclosporin (P-gP inhibitor). **(B) **Effects of JAI-51 on wild-type (S, sensitive) and on BCRP overexpressing (R, resistant) HCT116 cell lines, with or without mitoxantrone (BCRP substrate) and/or cyclosporin (BCRP inhibitor). **(C) **Percentage of cells in the G2/M phase of the cell cycle after JAI-51 treatment. K562/S and K562/R **(A)**, HCT1162/S and HCT116/R **(B) **cells were treated with DMSO (vehicle/0.1%) (control) or JAI-51 (10 μM) and analyzed as described in the Methods section. Drug accumulation results are expressed in mean fluorescence intensity (M.F.I.) in arbitrary units. The results are expressed as the mean ± SD from 3 different experiments. * *P *< 0.05 cyclosporin or JAI-51-treated resistant cells versus their respective control. **(C) **To test whether JAI-51 was a substrate of P-gp and/or BCRP, multidrug resistant cells and their respective parental cell lines (HCT116/R, HCT116/S, K562/R and K562/S) were incubated in the presence of JAI-51 (10 μM). Phases of the cell cycle were analyzed at 24 h as described in Figure 1B. G2/M is expressed in % of cells. The results are expressed as the mean ± SD from 3 different experiments. * *P *< 0.05 JAI-51-treated versus control.

In K562/R and HCT116/R cell lines, 10 μM JAI-51 restored the intracellular accumulation of daunorubicine (Figure 3/A) or mitoxantrone (Figure 3/B) with the same rate as the P-gp and BCRP-inhibitor cyclosporine A, showing that JAI-51 is also an inhibitor of these pumps. Figure 3/C shows that 10 μM JAI-51 was able to induce a cell cycle arrest in the G2/M phase, and an increase in both the percentage of apoptotic cells and the mitotic index (data not shown) of K562/R and S and HCT116/R and S cells. As the same effect of JAI-51 on the cell cycle was observed on all the cell lines tested, irrespective of the presence or not of an efflux pump, this shows that JAI-51 is not extruded by (i.e. is not a substrate of) P-gp (K562R cells) nor of BCRP (HCT116R cells). Taken together, these results show that JAI-51 is an inhibitor of P-gp and BCRP but is not a substrate of these pumps.

### Effect on tubulin polymerization and interaction tubulin-JAI-51

**Figure 4:**
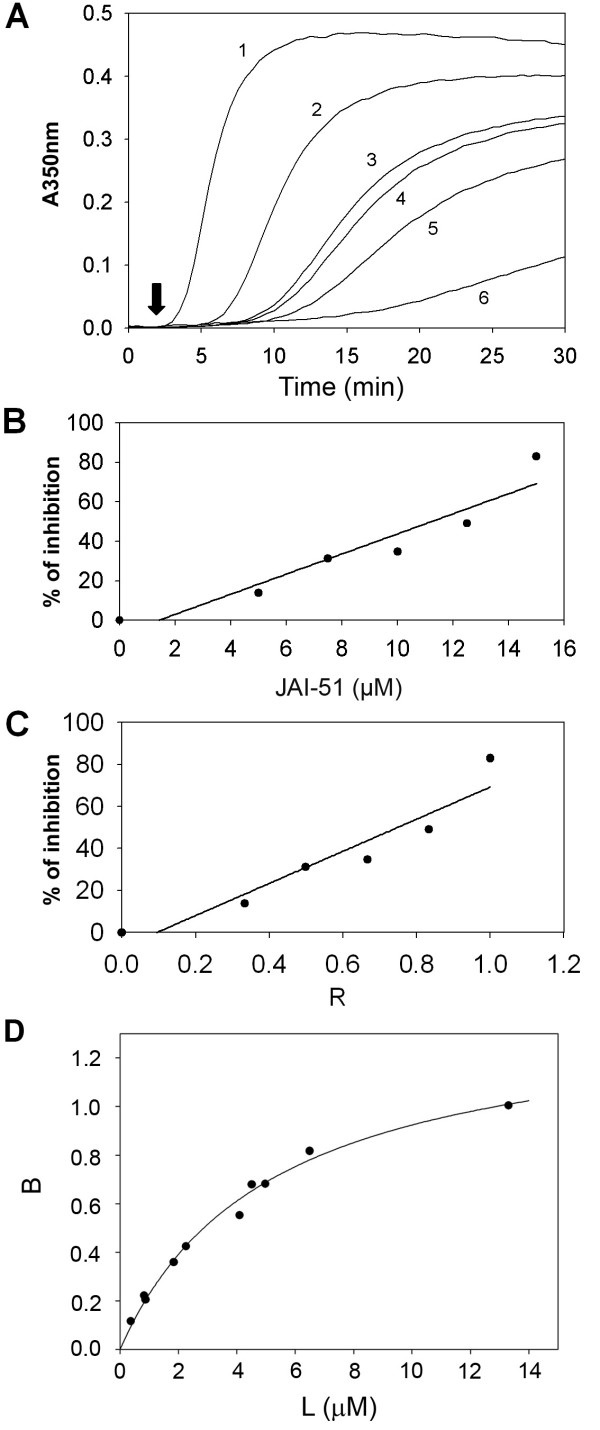
**Tubulin assembly in the presence of JAI-51 and binding of JAI-51 on tubulin**. **(A) **Effect of various concentrations of JAI-51 (1: 0 μM; 2: 5 μM; 3: 7.5 μM; 4: 10 μM; 5: 12.5 μM; 6: 15 μM) on 15 μM tubulin, in polymerization buffer. Assembly was started by warming the samples at 37°C (arrow). **(B) **Represents the fraction of the reduction of the plateau absorbance values (inhibition) as a function of total JAI-51 concentrations. **(C) **Percentage of polymerization inhibition as a function of the mole ratio of the total JAI-51 to total tubulin in the solution (R). **(D) **Study of the binding of JAI-51 on tubulin by analytical ultracentrifugation. Circles are the experimental points and the solid line is the fit obtained as described in the Methods section. In these experiments one binding site was found (n = 1.3 ± 0.6) with an affinity constant of (1.9 ± 0.6) 10^5 ^M^-1^.

Figure 4/A shows the effects of JAI-51 on the turbidimetry time course of microtubule assembly from pure tubulin. A clear inhibition was noted and the rate of assembly, as well as the final amount of microtubules, was lower in the presence of JAI-51 (curves 2–6) than in the control (curve 1). Figure 4/B shows that the turbidity generated by the self-assembly of 15 μM tubulin was reduced by half with 11.25 μM JAI-51. Figure 4/C shows that the extent of inhibition by JAI-51 increased monotonically with the mole ratio of the total ligand to total tubulin in the solution (R). In this figure, 50% inhibition occurred with 0.75 mol of JAI-51 per mol of tubulin, indicating a substoichiometric mode of inhibition.

To understand the mechanism of this inhibition, the interaction of JAI-51 with tubulin was studied by analytical ultracentrifugation. In the presence of JAI-51, the sedimentation coefficient of tubulin remained unchanged (5.4 S). Bound ligand concentration divided by total protein concentration (bound fraction, B) was plotted versus free ligand concentration (L) (Figure 4/D) and analyzed as described in the Methods section. JAI-51 displayed a stoichiometry of n = 1.3 ± 0.6 with an apparent association constant of (1.9 ± 0.6) × 10^5 ^M^-1^, similar to that of MTC (2methoxy-5-(2', 3', 4'-trimethoxyphenyl)-topone), a bifunctional analog of colchicine [13], with a structure closely related to that of MDL 27048 [trans-1-(2,5-dimethoxyphenyl)-3-[4-(dimethylamino)phenyl]-2-methyl-2-propen-1-one]. As these two latter compounds bind to the colchicine site, we examined the interaction of JAI-51 with tubulin-colchicine complexes by analytical ultracentrifugation. A stoichiometry of n = 0.40 ± 0.07 was measured, showing that the binding of JAI-51 to tubulin-colchicine complexes was essentially inhibited.

### *In vivo *effects in mice

**Figure 5:**
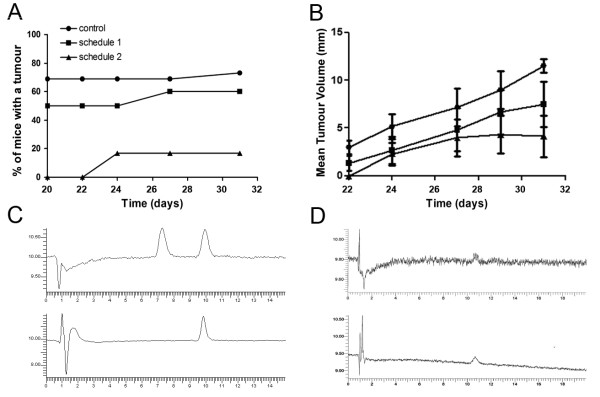
***In-vivo *effects of JAI-51 on mice bearing subcutaneous GL26 xenografts**. **(A) **Tumour onset and progression and **(B) **Mean tumour volumes, in JAI-51 treated GL26 xenografted mice. Control: untreated mice (n = 16), schedule 1: mice treated 3 times a week with 32 mg/kg JAI-51 (n = 10); schedule 2: mice treated once a week with 96 mg/kg JAI-51 (n = 6). Results are from one representative experiment out of two. For **(B) **statistical analysis using Dunn's Multiple Comparison Test following one-way ANOVA showed that the mean tumour volumes were significantly lower in the group treated with schedule 2 compared to the control group throughout the length of the experiment (*P *< 0.05). **(C, upper panel) **chromatogram of JAI-51 in the tumours of mice treated with schedule 2, an hour and a half before sacrifice. **(D, upper panel) **chromatogram of brain homogenates from JAI-51 treated mice. As controls, known amounts of JAI-51 were analyzed in the same experimental conditions **(C and D, lower panels)**.

In our murine xenograft model, the first tumours were observed on day 20 and the apparition of the tumours was monitored on days 22, 24, 27 and 31. Data in Figure 5/A show that the tumour onset and progression were delayed in the group of mice treated with schedule 1, and even more dramatically so in the group treated with schedule 2. Indeed, with schedule 2 (96 mg/kg JAI-51 once a week), the first tumours were detected on day 24 and the percentage of mice with a detectable tumour remained unchanged until the end of the experiment. In the group treated with schedule 1 (32 mg/kg three times a week), the number of mice with a detectable tumour and tumour progression was decreased compared to the control group, but to a lesser extent. Moreover, the mean tumour volumes were significantly lower in the group treated with schedule 2, throughout the length of the experiment (Figure 5/B) compared to the control group (*P *< 0.05) whereas there was no significant difference between the group treated with schedule 1 and the controls.

JAI-51 was detected by HPLC in the tumours of mice treated with schedule 2 an hour and a half before sacrifice (Figure 5/C, **upper panel**). As a control, a known concentration of JAI-51 was analyzed in the same experimental conditions as the samples to be sure that the peak detected corresponded to JAI-51 (Figure 5/C, **lower panel **and Figure 5/D, **lower panel**). The chromatograms showed a high intensity of JAI-51 in the tumours, ten times higher than the limit of detection which was 3.85 10^-7 ^M by this method (Figure 5/D, **lower panel**). A second peak was also detected, probably corresponding to a metabolite of the title compound.

JAI-51 was also detected by HPLC in the brain of xenografted mice treated following schedule 1 (Figure 5/D, **upper panel**). The same results were obtained in the brains of mice treated with schedule 2 (data not shown). JAI-51 was also detected in the liver and kidney of treated mice (data not shown); the yield of extraction (see the Material and Methods section for details) of the organs from treated mice, were 92 ± 4% in the brain and 89 ± 2 and 86 ± 3 in the kidney and liver respectively.

## Discussion

Flavonoid and chalcone derivatives possess various properties such as inhibition of cell proliferation, associated or not to an inhibition of tubulin polymerization [19], or modulation of ABC pumps [10,11,20]. Some of these molecules cross the BBB [9] and induce apoptosis in cancer cells [21], but to date no molecule displaying all these activities has been described. In this context, we report the *in-vitro *and *in-vivo *effects of a new chalcone derivative, named JAI-51.

*In vitro*, JAI-51 reduces the proliferation of glioblastoma cell lines from human and mouse origins and induces apoptosis, in some cases through a Caspase-3 dependent pathway, depending on the cell line studied. For U138 cell line, a Caspase-independent apoptosis process could be involved, as reviewed by Tait and Green [22]. This phenomenon can occur during mitosis and has been described to be a mechanism for protecting cells from an endoreplicative process [23]. It has also been demonstrated that flavonoids can induce apoptosis by both Caspase-mediated or Caspase-independent processes [24]. Our compound acts in a dose- or a time-dependent manner: low doses or short induction times induce a cytostatic effect whereas higher concentrations or higher induction times induce a cytolytic effect. The differences in the reactions of the various cell lines on JAI-51 incubation were not related to the presence of P-gp or BCRP, as a functional assay showed no accumulation of daunorubicin (P-gp substrate) or mitoxantrone (BCRP substrate) in these cell lines (data not shown). Hence, the differences in the cytostatic effects of JAI-51 on the cell lines tested were probably due to differences in the doubling times of these cell lines. Cytological analyses showed that JAI-51 blocks cell cycle in metaphase, probably by modulating microtubule stability. This destabilizing effect was characterized in a cell-free system and the substoichiometric concentrations of JAI-51 in relation with the tubulin concentration were sufficient to block tubulin polymerization in a way similar to vincaalkaloids or colchicine. JAI-51 is able to compete with colchicine for the same binding site that induces inhibition of tubulin polymerization. Tubulin targeting agents are usually classified in two groups, according to their effects: microtubule stabilizers such as taxol and epothilone and microtubule destabilizers including vincaalkaloids and colchicine. In the same way as microtubule destabilizers [25-27], JAI-51 is able to disrupt the formation of microtubules, leading to pronounced G2/M cell cycle arrest and apoptosis. Classically, one of the limiting phenomena for the use of common microtubule destabilizers is drug resistance mediated by P-gp or BCRP. However, in the present study, no differential cytotoxic activities (through cell cycle analysis) were observed between sensitive and resistant cell lines overexpressing P-gp or BCRP, suggesting that JAI-51 is not a substrate for these efflux pumps, in drastic contrast to vinblastine and paclitaxel [28]. In accordance with our results, Liu *et al*. [20] reported a chalcone which can inhibit both P-gp and BCRP and whose structure is similar to that of JAI-51, which was developed from a basic chalcone with a meta dimethoxy motif on ring A, an important feature for activity, as proposed by Seelig and Landwojtowicz [29]. Moreover, the active concentrations used in the study of Liu *et al*. [20] are closely related to those obtained in our experiments. However the authors didn't investigate the biological activities of their compound other that P-gp and BCRP inhibition.

*In vivo*, in a model of sc GL26 xenografted C57BL/6 mice, our compound induced a delay in tumour onset and a tumour growth inhibition at 96 mg/kg once a week (schedule 2). In accordance with these results, HPLC analysis of tumours from mice treated with schedule 2 showed that JAI-51 reached concentrations similar to those inducing a cytostatic effect in our *in vitro *studies.

JAI-51 was also detected by HPLC in the liver, kidney and brain of treated mice. As these organs express a high level of ABC pumps, these results reinforce those obtained *in vitro *showing that our compound is a modulator without being a substrate of these ABC pumps. Moreover, as JAI-51 was detected in the brain of the treated mice, this shows that our compound is able to cross the BBB. In addition, no toxicity in terms of blood cell counts, body weight or behaviour was observed in the treated animals (data not shown).

## Conclusion

In view of our results, when compared to other anticancer agents, our compound possesses a number of interesting properties. Alone or associated to recent progress made in the treatment of tumours of the CNS including either new methods for direct delivery of chemotherapy [30] or novel approaches for transporting drugs into the CNS [31], JAI-51 could be a promising new drug. In glioblastoma patients, JAI-51 could also be efficient on the malignant cells infiltrating the surrounding tumour and which are located under areas with an intact BBB [32].

## Competing interests

The authors declare that they have no competing interests.

## Authors' contributions

AB performed, supervised and designed studies on organic synthesis and analytical studies. AM-F performed *in vitro *cell biology and *in vivo *research, supervised experimental studies, wrote and made final changes to the manuscript. PC performed *in vitro *cell biology research and wrote part of the manuscript. DA performed *in vitro *tubulin polymerization assays. DM performed the extraction of the active compound from animal organs and HPLC analyses. FS performed and supervised HPLC analyses. VP designed research and wrote part of the manuscript dealing with *in vitro *tubulin polymerization assays. MD and BT designed and performed *in vivo *experiments. JB performed research, conceived the study, supervised its design and coordination, and made final changes to the manuscript.

All authors read and approved the final manuscript.

## Pre-publication history

The pre-publication history for this paper can be accessed here:

http://www.biomedcentral.com/1471-2407/9/242/prepub
